# Peripapillary pachychoroid syndrome: clinical insights

**DOI:** 10.1038/s41433-024-03274-z

**Published:** 2024-07-31

**Authors:** Peter Kiraly, M. Dominik Fischer

**Affiliations:** 1 Oxford Eye Hospital, Oxford University Hospitals NHS Foundation Trust, Oxford, UK; 2 Nuffield Laboratory of Ophthalmology, Nuffield Department of Clinical Neurosciences, University of Oxford, Oxford, UK; 3 University Eye Hospital Tübingen, University of Tübingen, Tübingen, Germany

**Keywords:** Retinal diseases, Anatomy

There is no consensus in the literature on whether peripapillary pachychoroid syndrome (PPS) is a separate entity within the pachychoroid disease spectrum or a subset of central serous chorioretinopathy (CSC) [1, 2]. A distinct clinical hallmark of PPS, which differentiates it from CSC, is the presence of intraretinal cysts that originate from the edge of the optic nerve disc and extend into the retina. In CSC, intraretinal cysts very rarely develop and are reported to be associated with prolonged chronic episodes (lasting more than five years) and subretinal fibrosis [3]. However, even these intraretinal cysts reported to be associated with CSC could instead be caused by secondary choroidal neovascularization (CNV), rather than CSC itself. In the absence of intraretinal cysts, PPS would just be CSC with sectoral choroidal thickening and subretinal fluid around the optic nerve disc.

In healthy individuals, there are no anastomoses between vortex vein systems, which appear as watershed zones on indocyanine green angiography. Spaide et al. showed that intervortex venous anastomoses develop in CSC patients in the macula, while in PPS patients they develop around the optic nerve disc [4], which leads to choroidal thickening, pachyvessels, hyperpermeability, and increased hydrostatic pressure in the choroid [5]. In CSC, increased hydrostatic pressure in the choroid leads to pigment epithelium detachment(s) (PED) and/or retinal pigment epithelium (RPE) bumps, through which leakage is observed in the subretinal space on fluorescein angiography. On the other hand, the majority of PPS patients do not show any leakage with only mild leakage observed in only few cases [1]. We believe therefore, that the formation of intraretinal cysts in PPS might be caused by gentle oozing of fluid from the peripapillary choroid instead of the high-flow leakage observed in CSC. While normal anatomy with an intact barrier function of the RPE would prevent significant intraretinal cyst formation under low-flow conditions, a pre-disposition with peripapillary RPE atrophy would support our conclusion. In the vast majority (if not all) of our PPS patients, we have observed peripapillary atrophy (Figs. 1–2), which is seen but not necessarily described in figures of previous PPS manuscripts [1, 2, 6]. Therefore, peripapillary RPE atrophy might be a predisposing alteration and not a consequence of long-term intraretinal fluid in PPS. As no PEDs with subretinal fluid accumulation were noted in the majority of our PPS patients, we hypothesize that hydrostatic choroidal pressure in PPS is not significant enough to cause CSC but sufficient to cause intraretinal cysts in cases with peripapillary atrophy. This is supported by the very mild leakage observed on angiography in PPS. Moreover, the peripapillary fluid pocket that develops in the area of peripapillary atrophy, leading to intraretinal fluid extension into more inner retinal layers distally, is a strong clue of intraretinal fluid development in PPS patients. This is seen in our patient in Fig. 1 and in previous manuscripts as well [2, 6]. PPS patients can also exhibit multiple areas of leakage; leakage through peripapillary atrophy contributes to intraretinal cysts, while PED/RPE bumps lead to subretinal fluid accumulation. Regarding differential diagnosis, peripapillary CNV and optic disc pitmaculopathy can closely resemble PPS on optical coherence tomography (OCT), with the distinction being the presence of fibrovascular PED in peripapillary CNV and a pit in optic disc pitmaculopathy.

**Fig. 1:**
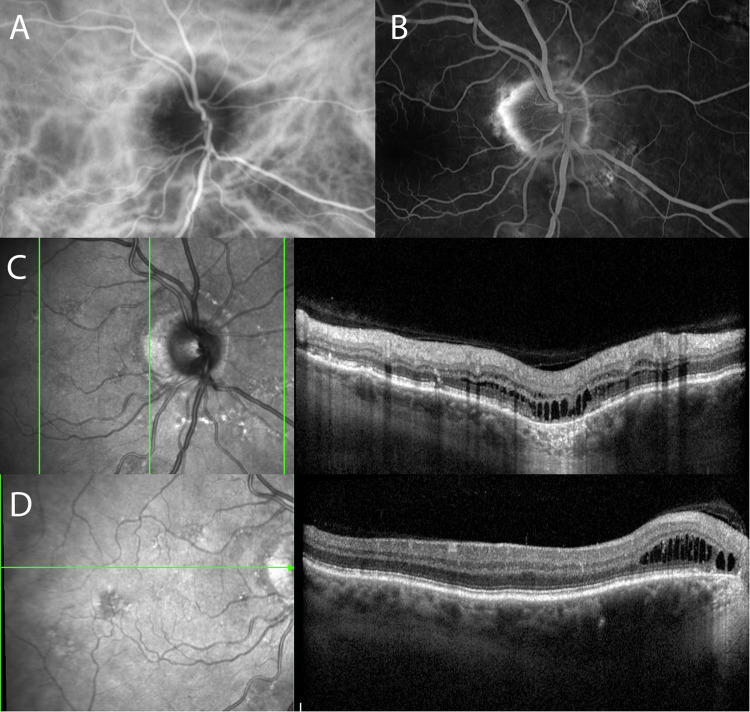
Multimodal imaging in a patient with peripapillary pachychoroid syndrome. (**A**) Several inter-vortex anastomoses around the optic nerve disc observed on indocyanine green angiography; (**B**) Mild leakage temporal to the optic nerve disc in the area of peripapillary atrophy seen on fluorescein angiography; (**C**) Vertical and (**D**) horizontal optic coherence tomography (OCT) scans showing intraretinal cysts originating from peripapillary atrophy, leading to intraretinal fluid extension distally.

**Fig. 2:**
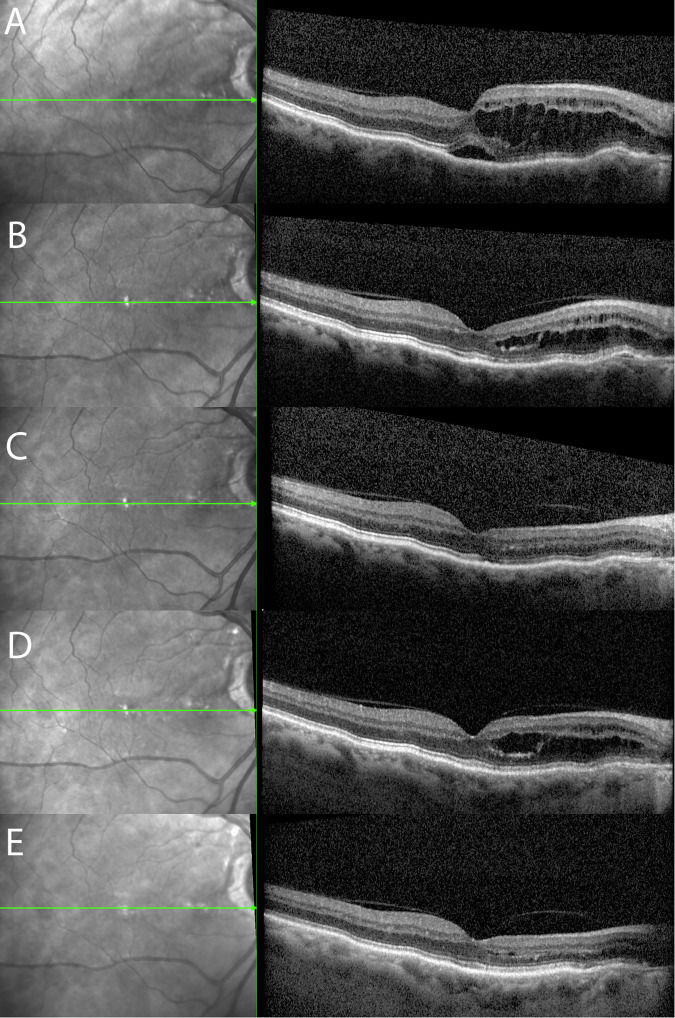
Long-term follow-up of a patient with peripapillary pachychoroid syndrome reveals spontaneous intraretinal fluid fluctuations. Serial foveal optical coherence tomography (OCT) scans: (**A**) baseline; (**B**) 20 months post-baseline; (**C**) 30 months post-baseline; (**D**) 57 months post-baseline; (**E**) 64 months post-baseline.

In PPS, intraretinal cysts originate at the optic disc margin and propagate temporally towards the fovea, with subfoveal subretinal fluid usually forming when cysts reach the fovea. With foveal involvement, patients usually become symptomatic. PPS patients are often asymptomatic and referred by a community optometrist due to incidental findings on OCT. In CSC, subretinal fluid accumulation follows a waxing and waning course [7]. In our PPS patients (Fig. 2), we observed fluctuations in a similar fashion. However, instead of subretinal fluid, we noted fluctuations in intraretinal fluid, which could follow changes in choroidal hydrostatic pressure. Therefore, PPS patients with extrafoveal intraretinal cysts could be monitored only. Even PPS patients with foveal involvement of short duration might be observed in a similar fashion to acute CSC patients due to possible spontaneous resolution. However, future prospective studies will need to confirm this approach as acceptable. Recently, several studies and case reports have highlighted topical steroids as a treatment option for PPS [8–10]. This approach seems unusual for treating patients within the pachychoroid spectrum, as increased endogenous and exogenous steroids have been clearly shown as the most important risk factor and could lead to more severe episodes [11]. These publications lack a control group, and reported improvements with steroid treatment could merely follow the natural course of the condition [12]. Several previous high-quality studies have established photodynamic therapy (PDT) as a gold standard for treating conditions within the pachychoroid spectrum, such as CSC [7]. A smaller study also confirmed PDT treatment as a safe and effective option for PPS [13]. Therefore, we recommend treating selected PPS patients with PDT instead of other treatment modalities.

## Data Availability

In this manuscript, all data generated or analysed are included.
